# Author Correction: VGLL1 cooperates with TEAD4 to control human trophectoderm lineage specification

**DOI:** 10.1038/s41467-025-57929-w

**Published:** 2025-03-18

**Authors:** Yueli Yang, Wenqi Jia, Zhiwei Luo, Yunpan Li, Hao Liu, Lixin Fu, Jinxiu Li, Yu Jiang, Junjian Lai, Haiwei Li, Babangida Jabir Saeed, Yi Zou, Yuan Lv, Liang Wu, Ting Zhou, Yongli Shan, Chuanyu Liu, Yiwei Lai, Longqi Liu, Andrew P. Hutchins, Miguel A. Esteban, Md. Abdul Mazid, Wenjuan Li

**Affiliations:** 1 State Key Laboratory for Diagnosis and Treatment of Severe Zoonotic Infectious Diseases, Key Laboratory for Zoonosis Research of the Ministry of Education, Institute of Zoonosis, and College of Veterinary Medicine, Jilin University, Changchun, China; 2 Laboratory of Integrative Biology, Guangzhou Institutes of Biomedicine and Health, Chinese Academy of Sciences (CAS), Guangzhou, China; 3 CAS Key Laboratory of Regenerative Biology and Guangdong Provincial Key Laboratory of Stem Cells and Regenerative Medicine, Guangzhou Institutes of Biomedicine and Health, Guangzhou, China; 4 University of Chinese Academy of Sciences, Beijing, China; 5 BGI Research, Shenzhen, China; 6 Joint School of Life Sciences, Guangzhou Institutes of Biomedicine and Health, Chinese Academy of Sciences, Guangzhou Medical University, Guangzhou, China; 7 Stem Cell Research Facility, Sloan Kettering Institute, New York, NY USA; 8 BGI Research, Hangzhou, China; 9 College of Life Sciences, University of Chinese Academy of Sciences, Beijing, China; 10 Department of Systems Biology, School of Life Sciences, Southern University of Science and Technology, Shenzhen, China

Correction to: *Nature Communications* 10.1038/s41467-024-44780-8, published online 17 January 2024

In the version of the article initially published, there were duplication errors in Supplementary Fig. 3h (duplicate of Supplementary Fig. 3n), Supplementary Fig. 3q (duplicate of Supplementary Fig. 2h), Supplementary Fig. 4h (duplicate of Supplementary Fig. 3p), Supplementary Fig. 7v (duplicate of Supplementary Fig. 7w), Supplementary Fig. 8m (the TFAP2c blot for the VGLL1 sample was a duplicate of the adjacent TFAP2C blot in GATA3 sample), while two blots in Supplementary Fig. 9 were from a different set of experiments. For comparison, the original and revised images are available in the Supplementary information accompanying this amendment. The mistakes arose during figure preparation and are errors in representation, only, and do not impact the conclusions of the study. The Supplementary Figs. are now updated in the HTML version of the article.

**Supplementary information**

Original and revised Supplementary Figs. 3h, 3q, 4h, 7v, 8m, 9.

## Supplementary information

Supplementary information

