# Supplementary material for: VGLL1 cooperates with TEAD4 to control human trophectoderm lineage specification
Source: Nat Commun. 2024 Jan 17;15:583. doi: 10.1038/s41467-024-44780-8 (PMC10794710; doi:10.1038/s41467-024-44780-8)
Supplement: Supplementary file 1 — Supplementary Information [file 41467_2024_44780_MOESM1_ESM.pdf]

Supplementary information for

VGLL1 cooperates with TEAD4 to control human trophectoderm lineage specification

Yueli Yang[1,11], Wenqi Jia[2,3,4,11], Zhiwei Luo[2,3], Yunpan Li[2,3], Hao Liu[2,3], Lixin Fu[4,5], Jinxiu Li[4,5], Yu Jiang[1], Junjian Lai[2,3,5], Haiwei Li[6], Babangida Jabir Saeed[2,3], Yi Zou[5], Yuan Lv[2,3,5], Liang Wu[2,3], Ting Zhou[7], Yongli Shan[3], Chuanyu Liu[5], Yiwei Lai[5,8], Longqi Liu[5,8,9], Andrew P. Hutchins[10], Miguel A. Esteban[1,2,3,5,8]✉, Md. Abdul Mazid[2,3]✉ and Wenjuan Li[2,3]✉

✉email: miguel@gibh.ac.cn; mazid@gibh.ac.cn; li_wenjuan@gibh.ac.cn

File list:

Supplementary figures

Supplementary Fig. 1

Supplementary Fig. 2

Supplementary Fig. 3

Supplementary Fig. 4

Supplementary Fig. 5

Supplementary Fig. 6

Supplementary Fig. 7

Supplementary Fig. 8

Supplementary Fig. 9

Supplementary tables

Supplementary Table 1

Supplementary Table 2

Supplementary Table 3

Supplementary Table 4

Supplementary Table 5

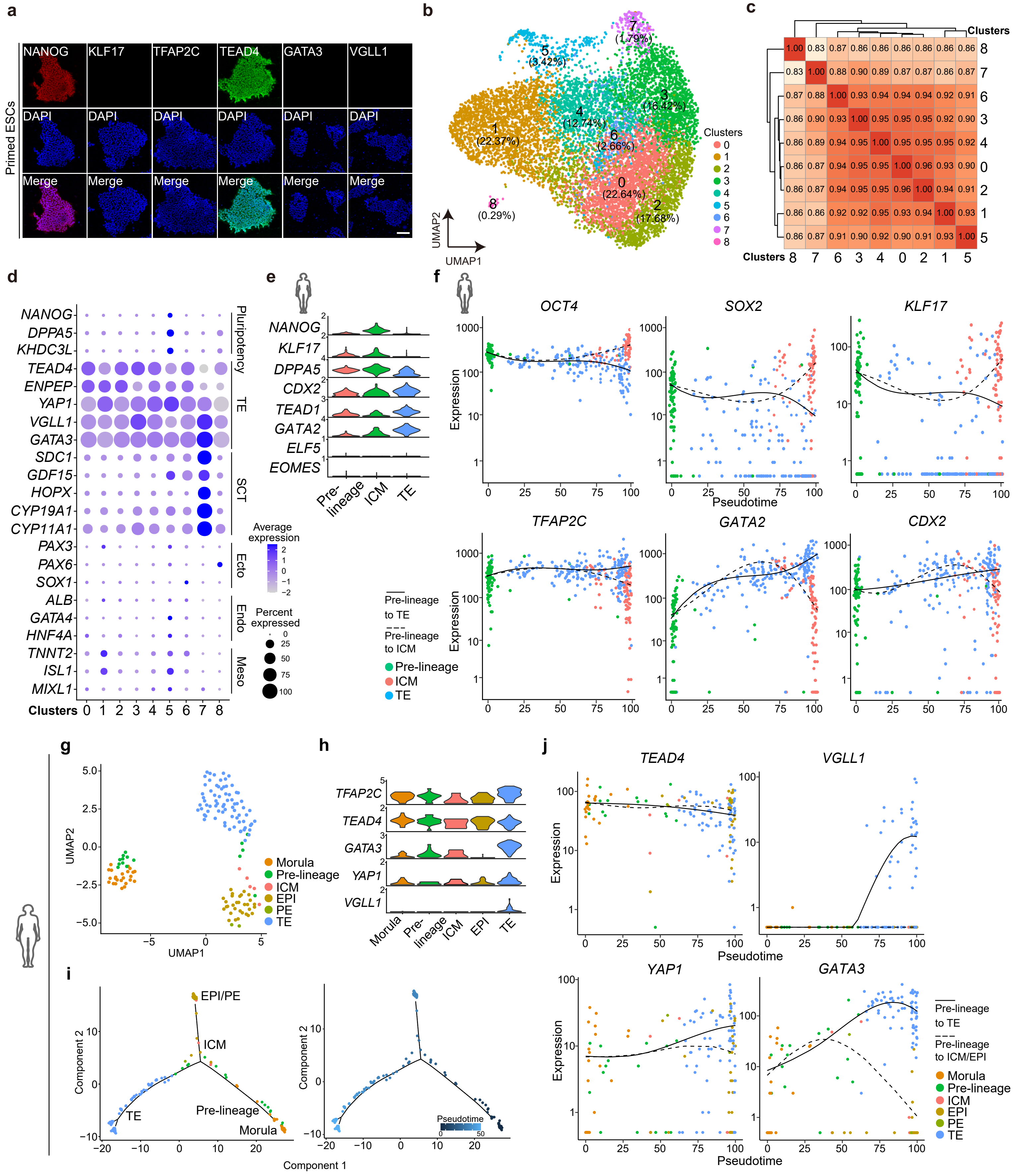

Supplementary Fig. 1

Supplementary Fig. 1. VGLL1 is highly and specifically expressed in human TE lineage.

a. Immunostaining images for pluripotency (NANOG and KLF17), naïve/TE shared (TFAP2C and TEAD4), and TE-enriched (GATA3 and VGLL1) genes in H9 primed ESCs (scale bar, 100 μm). Nuclei were counterstained with DAPI (blue). Representative of three independent experiments.

b. UMAP visualization of 4CL H9 ESC-derived TELC-D5 cells. Cells are colored by unsupervised clustering analysis.

c. Pseudobulk Pearson correlation analysis of the different clusters in 4CL H9 ESC-derived TELC-D5 cells of panel b.

d. Bubble plot representing the frequency of expression and scaled average expression of representative genes in each cluster of 4CL H9 ESC-derived TELC-D5 cells. Ecto: ectoderm. Endo: endoderm. Meso: mesoderm.

e. Violin plot showing the log-normalized expression of additional pluripotency and TE genes in different cell types of the human blastocyst at E5.

f. Expression patterns of additional pluripotency and TE genes along the pseudotime trajectory in the human blastocyst at E5.

g. UMAP visualization showing the different cell types in the human embryo E4-E6 stage[41]. Dots are colored by cell type. Cell types were annotated by cluster-specific gene expression patterns.

h. Violin plot showing the log-normalized expression of TE genes in different cell types of the human embryo E4-E6 stage.

i. Left panel: the trajectory of the different cell types in the human embryo E4-E6 stage reveals two branches: pre-lineage to the TE branch and pre-lineage to the ICM/EPI branch. Right panel: colors from dark blue to light blue indicate progression through the pseudotime.

j. Expression patterns of TE genes along the pseudotime trajectory of cells in the human embryo E4-E6 stage.

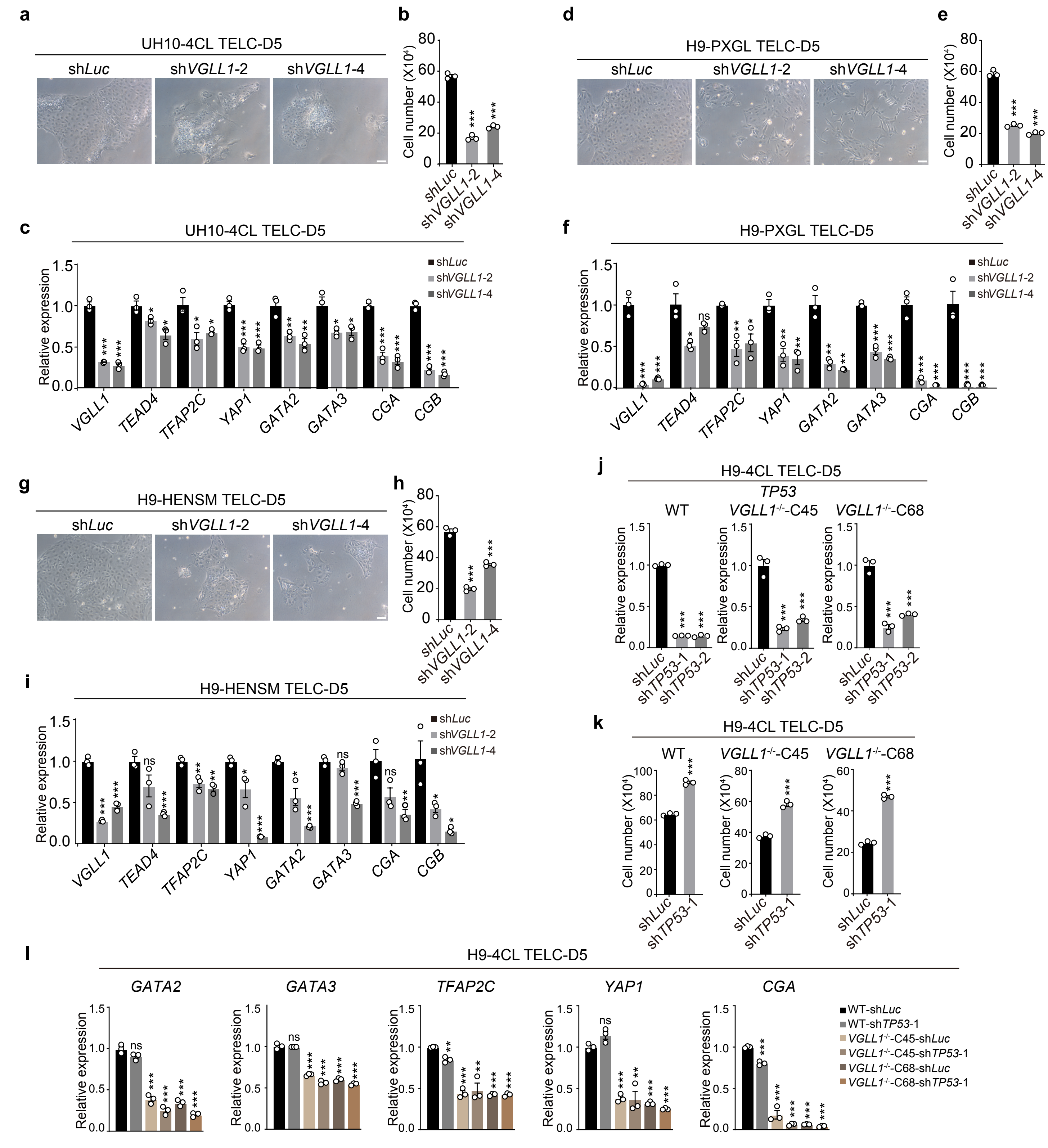

Supplementary Fig. 2

Supplementary Fig. 2. VGLL1 is indispensable for TELC induction in different naïve PSCs and cell lines.

a. Representative phase contrast images of 4CL UH10 iPSCs transduced with sh*Luc* (control) or sh*VGLL1* (2 and 4) at day 5 of TELC differentiation. Scale bar, 100 μm. Representative of three independent experiments.

b. Analysis of cell numbers for 4CL UH10 iPSCs transduced with sh*Luc*, sh*VGLL1*-2 or sh*VGLL1*-4 at day 5 of TELC differentiation. Data are presented as the mean ± SEM. n=3 biological replicates. *P* value was calculated using a two-tailed unpaired Student's *t*-test, ****P* < 0.001.

c. RT-qPCR showing the expression of TE-related genes for 4CL UH10 iPSCs transduced with sh*Luc*, sh*VGLL1*-2 or sh*VGLL1*-4 at day 5 of TELC differentiation. Data are presented as the mean ± SEM. n=3 biological replicates. *P* value was calculated using a two-tailed unpaired Student's *t*-test, ****P* < 0.001, ***P* < 0.01, **P* < 0.05.

d. Representative phase contrast images of PXGL H9 ESCs transduced with sh*Luc*, sh*VGLL1*-2 or sh*VGLL1*-4 at day 5 of TELC differentiation. Scale bar, 100 μm. Representative of three independent experiments.

e. Analysis of cell numbers for PXGL H9 ESCs transduced with sh*Luc*, sh*VGLL1*-2 or sh*VGLL1*-4 at day 5 of TELC differentiation. Data are presented as the mean ± SEM. n=3 biological replicates. *P* value was calculated using a two-tailed unpaired Student's *t*-test, ****P* < 0.001.

f. RT-qPCR showing the expression of TE-related genes for PXGL H9 ESCs transduced with sh*Luc*, sh*VGLL1*-2 or sh*VGLL1*-4 at day 5 of TELC differentiation. Data are presented as the mean ± SEM. n=3 biological replicates. *P* value was calculated using a two-tailed unpaired Student's *t*-test, ****P* < 0.001, ***P* < 0.01, **P* < 0.05.

g. Representative phase contrast images of HENSM H9 ESCs transduced with sh*Luc*, sh*VGLL1*-2 or sh*VGLL1*-4 at day 5 of TELC differentiation. Scale bar, 100 μm. Representative of three independent experiments.

h. Analysis of cell numbers for HENSM H9 ESCs transduced with sh*Luc*, sh*VGLL1*-2 or sh*VGLL1*-4 at day 5 of TELC differentiation. Data are presented as the mean ± SEM. n=3

biological replicates. *P* value was calculated using a two-tailed unpaired Student's *t*-test, ***$P < 0.001$.

i. RT-qPCR showing the expression of TE-related genes for HENSM H9 ESCs transduced with sh*Luc*, sh*VGLL1*-2 or sh*VGLL1*-4 at day 5 of TELC differentiation. Data are presented as the mean ± SEM. n=3 biological replicates. *P* value was calculated using a two-tailed unpaired Student's *t*-test, ***$P < 0.001$, **$P < 0.01$, *$P < 0.05$.

j. RT-qPCR showing the *TP53* knockdown efficiency for WT H9 cells and *VGLL1*-KO cells transduced with sh*TP53*-1 or sh*TP53*-2 compared to the sh*Luc* control at day 5 of TELC differentiation. Data are presented as the mean ± SEM, n=3 biological replicates. *P* value was calculated using a two-tailed unpaired Student's *t*-test, ***$P < 0.001$.

k. Analysis of cell numbers for WT H9 cells and *VGLL1*-knockout cells transduced with sh*Luc* and sh*TP53*-1 at day 5 of TELC differentiation. Data are presented as the mean ± SEM. n=3 biological replicates. *P* value was calculated using a two-tailed unpaired Student's *t*-test, ***$P < 0.001$.

l. RT-qPCR showing the expression of TE-related genes for WT H9 cells and *VGLL1*-KO cells transduced with sh*Luc* and sh*TP53*-1 at day 5 of TELC differentiation. Data are presented as the mean ± SEM, n=3 biological replicates. *P* value was calculated using a two-tailed unpaired Student's *t*-test, ***$P < 0.001$, **$P < 0.01$, ns: not significant.

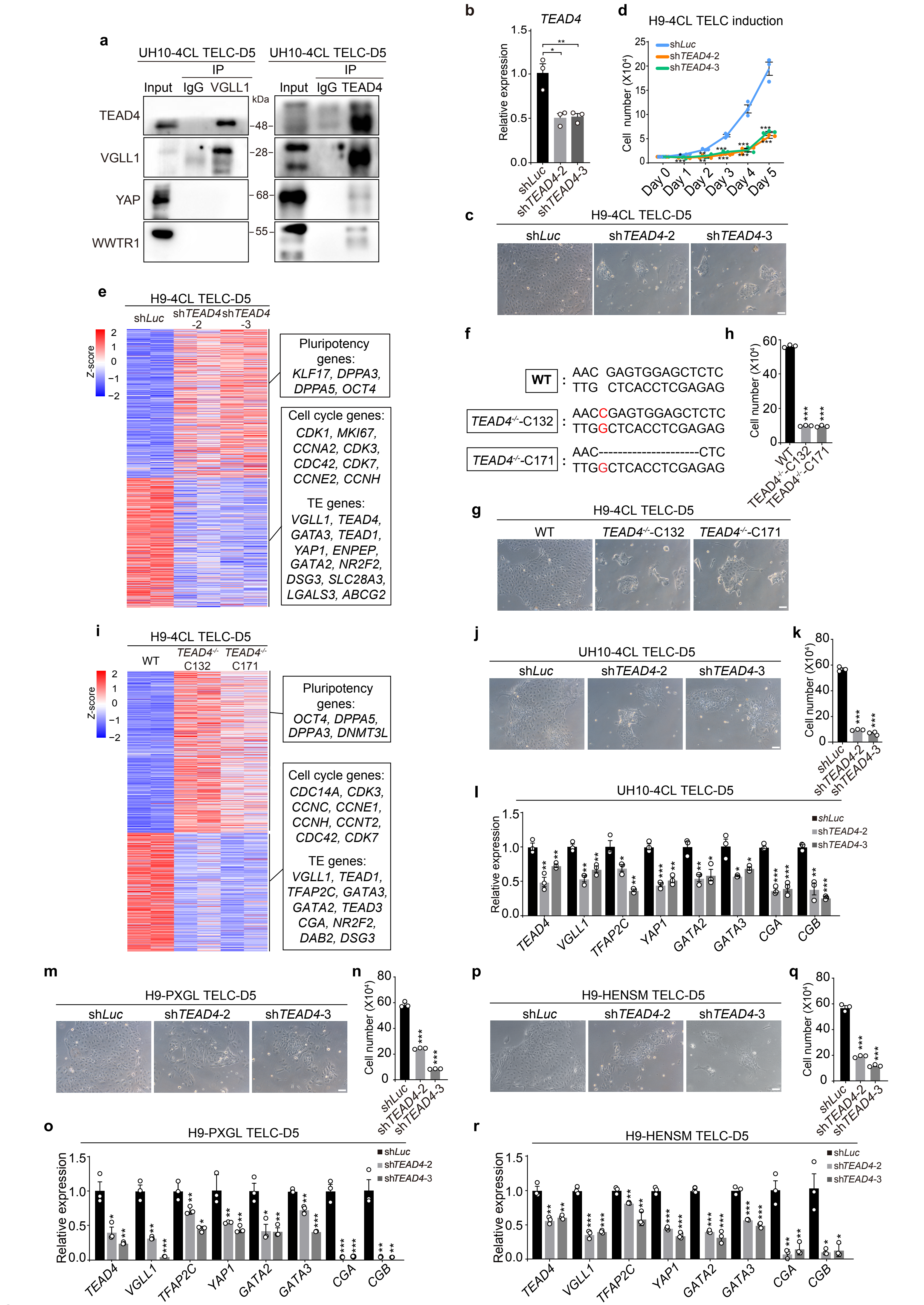

Supplementary Fig. 3

Supplementary Fig. 3. TEAD4 depletion impedes TELC induction in different naïve PSCs and cell lines.

a. Immunoprecipitation using lysates from TELC-D5 cells differentiated from 4CL UH10 iPSCs with anti-VGLL1 (left panel) or anti-TEAD4 (right panel) and subsequent Western blotting analysis with anti-TEAD4, anti-VGLL1, anti-YAP and anti-WWTR1. Representative of three independent experiments.

b. RT-qPCR showing the *TEAD4* knockdown efficiency for 4CL H9 ESCs transduced with sh*TEAD4*-2 or sh*TEAD4*-3 compared to sh*Luc* control at day 5 of TELC differentiation. Data are presented as the mean ± SEM, n=3 biological replicates. *P* value was calculated using a two-tailed unpaired Student's *t*-test, **P* < 0.05.

c. Phase contrast images of 4CL H9 ESCs transduced with sh*Luc*, sh*TEAD4*-2 or sh*TEAD4*-3 at day 5 of TELC differentiation. Scale bar, 100 μm. Representative of three independent experiments.

d. Analysis of cell numbers for 4CL H9 ESCs transduced with sh*Luc*, sh*TEAD4*-2 or sh*TEAD4*-3 along the TE induction time course. Data are presented as the mean ± SEM, n=3 biological replicates. *P* value was calculated using a two-tailed unpaired Student's t-test, ****P* < 0.001, ***P* < 0.01, **P* < 0.05.

e. Heatmap showing the expression of pluripotency, cell cycle- and TE genes at day 5 of TELC differentiation for the indicated conditions. Example genes are shown for each cluster in the boxes. n=2 biological replicates.

f. TA cloning followed by Sanger sequencing results showing homozygous deletion for *TEAD4*-KO clones [clone 132 (C132) and clone 171 (C171)]. WT: wild-type.

g. Representative phase contrast images of WT and *TEAD4*-KO H9 ESC clones at day 5 of TELC differentiation. Scale bar, 100 μm. Representative of three independent experiments.

h. Analysis of cell numbers for WT and *TEAD4*-KO H9 ESC clones at day 5 of TELC differentiation. Data are presented as the mean ± SEM. n=3 biological replicates. *P* value was calculated using a two-tailed unpaired Student's *t*-test, ****P* < 0.001.

i. Heatmap showing the expression of pluripotency, cell cycle and TE genes in bulk RNA-seq of H9 *TEAD4*-KO clones compared to WT at day 5 of TELC differentiation. Example genes are shown for each cluster in the boxes. n=2 biological replicates.

j. Phase contrast images of 4CL UH10 iPSCs transduced with sh*Luc*, sh*TEAD4*-2 or sh*TEAD4*-3 at day 5 of TELC differentiation. Scale bar, 100 μm. Representative of three independent experiments.

k. Analysis of cell numbers for 4CL UH10 iPSCs transduced with sh*Luc*, sh*TEAD4*-2 or sh*TEAD4*-3 at day 5 of TELC differentiation. Data are presented as the mean ± SEM. n=3 biological replicates. *P* value was calculated using a two-tailed unpaired Student's *t*-test, $^{***}P < 0.001$.

l. RT-qPCR showing the expression of TE genes for 4CL UH10 iPSCs transduced with sh*Luc*, sh*TEAD4*-2 or sh*TEAD4*-3 at day 5 of TELC differentiation. Data are presented as the mean ± SEM. n=3 biological replicates. *P* value was calculated using a two-tailed unpaired Student's *t*-test, $^{***}P < 0.001$, $^{**}P < 0.01$, $^{*}P < 0.05$.

m. Phase contrast images of PXGL H9 ESCs transduced with sh*Luc*, sh*TEAD4*-2 or sh*TEAD4*-3 at day 5 of TELC differentiation. Scale bar, 100 μm. Representative of three independent experiments.

n. Analysis of cell numbers for PXGL H9 ESCs transduced with sh*Luc*, sh*TEAD4*-2 or sh*TEAD4*-3 at day 5 of TELC differentiation. Data are presented as the mean ± SEM. n=3 biological replicates. *P* value was calculated using a two-tailed unpaired Student's *t*-test, $^{***}P < 0.001$.

o. RT-qPCR showing the expression of TE genes for PXGL H9 ESCs transduced with sh*Luc*, sh*TEAD4*-2 or sh*TEAD4*-3 at day 5 of TELC differentiation. Data are presented as the mean ± SEM. n=3 biological replicates. *P* value was calculated using a two-tailed unpaired Student's *t*-test, $^{***}P < 0.001$, $^{**}P < 0.01$, $^{*}P < 0.05$.

p. Phase contrast images of HENSM H9 ESCs transduced with sh*Luc*, sh*TEAD4*-2 or sh*TEAD4*-3 at day 5 of TELC differentiation. Scale bar, 100 μm. Representative of three independent experiments.

q. Analysis of cell numbers for HENSM H9 ESCs transduced with sh*Luc*, sh*TEAD4*-2 or sh*TEAD4*-3 at day 5 of TELC differentiation. Data are presented as the mean ± SEM. n=3 biological replicates. *P* value was calculated using a two-tailed unpaired Student's *t*-test, $^{***}P < 0.001$.

r. RT-qPCR showing the expression of TE genes for HENSM H9 ESCs transduced with sh*Luc*, sh*TEAD4*-2 or sh*TEAD4*-3 at day 5 of TELC differentiation. Data are presented as the mean ± SEM. n=3 biological replicates. *P* value was calculated using a two-tailed unpaired Student's *t*-test, ***$P < 0.001$, **$P < 0.01$, *$P < 0.05$.

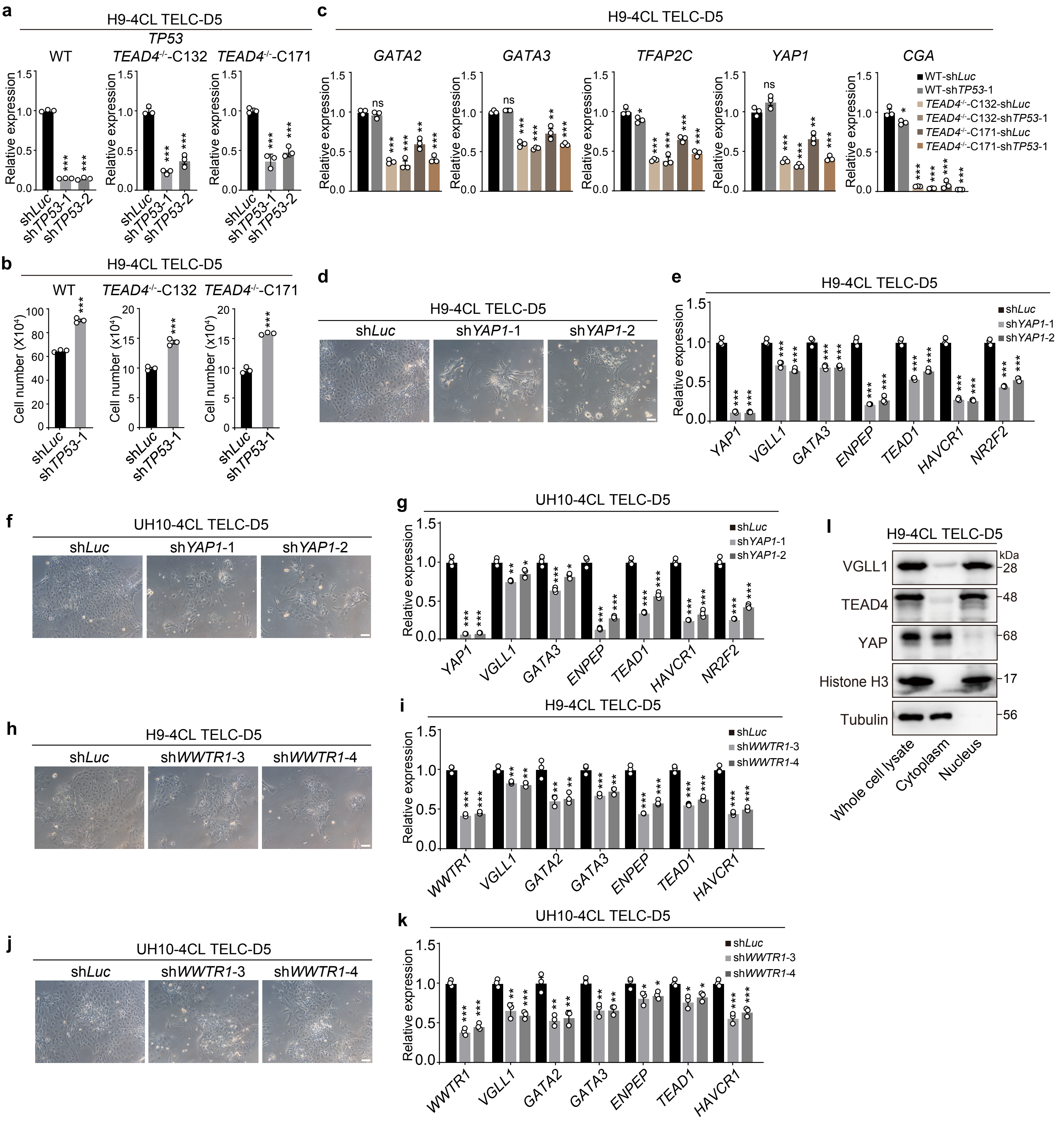

Supplementary Fig. 4

Supplementary Fig. 4. YAP1 and WWTR1 knockdown impairs human TELC induction.

a. RT-qPCR showing the *TP53* knockdown efficiency for WT and *TEAD4*-KO 4CL H9 ESCs transduced with sh*TP53*-1 or sh*TP53*-2 compared to the sh*Luc* control at day 5 of TELC differentiation. Data are presented as the mean ± SEM, n=3 biological replicates. *P* value was calculated using a two-tailed unpaired Student's *t*-test, ***$P < 0.001$.

b. Analysis of cell numbers for WT and *TEAD4*-KO H9 ESCs transduced with sh*Luc* and sh*TP53*-1 at day 5 of TELC differentiation. Data are presented as the mean ± SEM. n=3 biological replicates. *P* value was calculated using a two-tailed unpaired Student's *t*-test, ***$P < 0.001$.

c. RT-qPCR showing the expression of TE genes for WT and *TEAD4*-KO H9 ESCs transduced with sh*Luc* and sh*TP53*-1. Data are presented as the mean ± SEM, n=3 biological replicates. *P* value was calculated using a two-tailed unpaired Student's *t*-test, ***$P < 0.001$, **$P < 0.01$, *$P < 0.05$, ns: not significant.

d. Phase contrast images of 4CL H9 ESCs transduced with sh*Luc*, sh*YAP1*-1 or sh*YAP1*-2 at day 5 of TELC differentiation. Scale bar, 100 μm. Representative of three independent experiments.

e. RT-qPCR showing the expression of TE genes for 4CL H9 ESCs transduced with sh*Luc*, sh*YAP1*-1 or sh*YAP1*-2 at day 5 of TELC differentiation. Data are presented as the mean ± SEM. n=3 biological replicates. *P* value was calculated using a two-tailed unpaired Student's *t*-test, ***$P < 0.001$.

f. Phase contrast images of 4CL UH10 iPSCs transduced with sh*Luc*, sh*YAP1*-1 or sh*YAP1*-2 at day 5 of TELC differentiation. Scale bar, 100 μm. Representative of three independent experiments.

g. RT-qPCR showing the expression of TE genes for 4CL UH10 iPSCs transduced with sh*Luc*, sh*YAP1*-1 or sh*YAP1*-2 at day 5 of TELC differentiation. Data are presented as the mean ± SEM. n=3 biological replicates. *P* value was calculated using a two-tailed unpaired Student's *t*-test, ***$P < 0.001$, **$P < 0.01$, *$P < 0.05$.

h. Phase contrast images of 4CL H9 ESCs transduced with sh*Luc*, sh*WWTR1*-3 or sh*WWTR1*-4 at day 5 of TELC differentiation. Scale bar, 100 μm. Representative of three independent experiments.

i. RT-qPCR showing the expression of TE genes for 4CL H9 ESCs transduced with sh*Luc*, sh*WWTR1*-3 or sh*WWTR1*-4 at day 5 of TELC differentiation. Data are presented as the mean ± SEM. n=3 biological replicates. *P* value was calculated using a two-tailed unpaired Student's *t*-test, ***$P < 0.001$, **$P < 0.01$.

j. Phase contrast images of 4CL UH10 iPSCs transduced with sh*Luc*, sh*WWTR1*-3 or sh*WWTR1*-4 at day 5 of TELC differentiation. Scale bar, 100 μm. Representative of three independent experiments.

k. RT-qPCR showing the expression of TE genes for 4CL UH10 iPSCs transduced with sh*Luc*, sh*WWTR1*-3 or sh*WWTR1*-4 at day 5 of TELC differentiation. Data are presented as the mean ± SEM. n=3 biological replicates. *P* value was calculated using a two-tailed unpaired Student's *t*-test, ***$P < 0.001$, **$P < 0.01$, *$P < 0.05$.

l. Fractionation Western blotting analysis for the indicated proteins in TELC-D5 cells differentiated from 4CL H9 ESCs. Representative of three independent experiments.

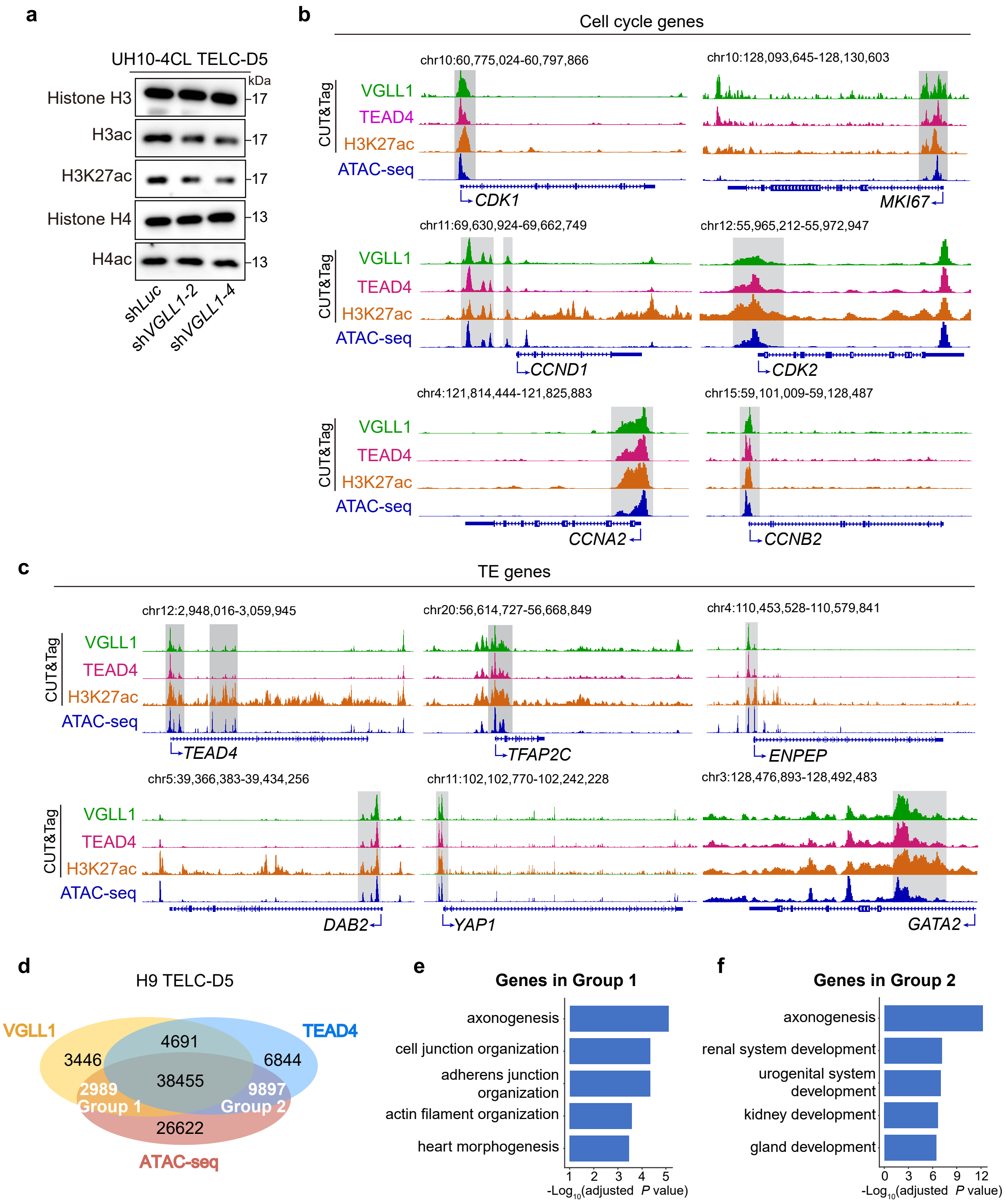

Supplementary Fig. 5

Supplementary Fig. 5. VGLL1/TEAD4/H3K27ac enrichment correlates with chromatin accessibility at target loci in human naïve PSC-derived TELCs .

a. Western blotting analysis for the indicated histone marks in 4CL UH10 iPSCs transduced with sh*Luc*, sh*VGLL1*-2 or sh*VGLL1*-4 at day 5 of TELC differentiation. Representative of three independent experiments.

b and **c**. Genome browser tracks showing VGLL1, TEAD4, H3K27ac genomic enrichment peaks (CUT&Tag) and chromatin accessibility peaks (ATAC-seq) for representative cell cycle- (**b**) and TE- (**c**) genes in TELC-D5 cells differentiated from 4CL H9 ESCs.

d. Venn diagram showing the overlap between VGLL1- and TEAD4-bound sites in CUT&Tag with chromatin accessibility peaks (ATAC-seq) in TELC-D5 cells differentiated from 4CL H9 ESCs.

e and **f.** Enriched GO terms for group 1 (**e**) and group 2 (**f**) genes of panel d. *P* value was calculated using a hypergeometric test and adjusted for multiple testing using the Benjamini-Hochberg correction.

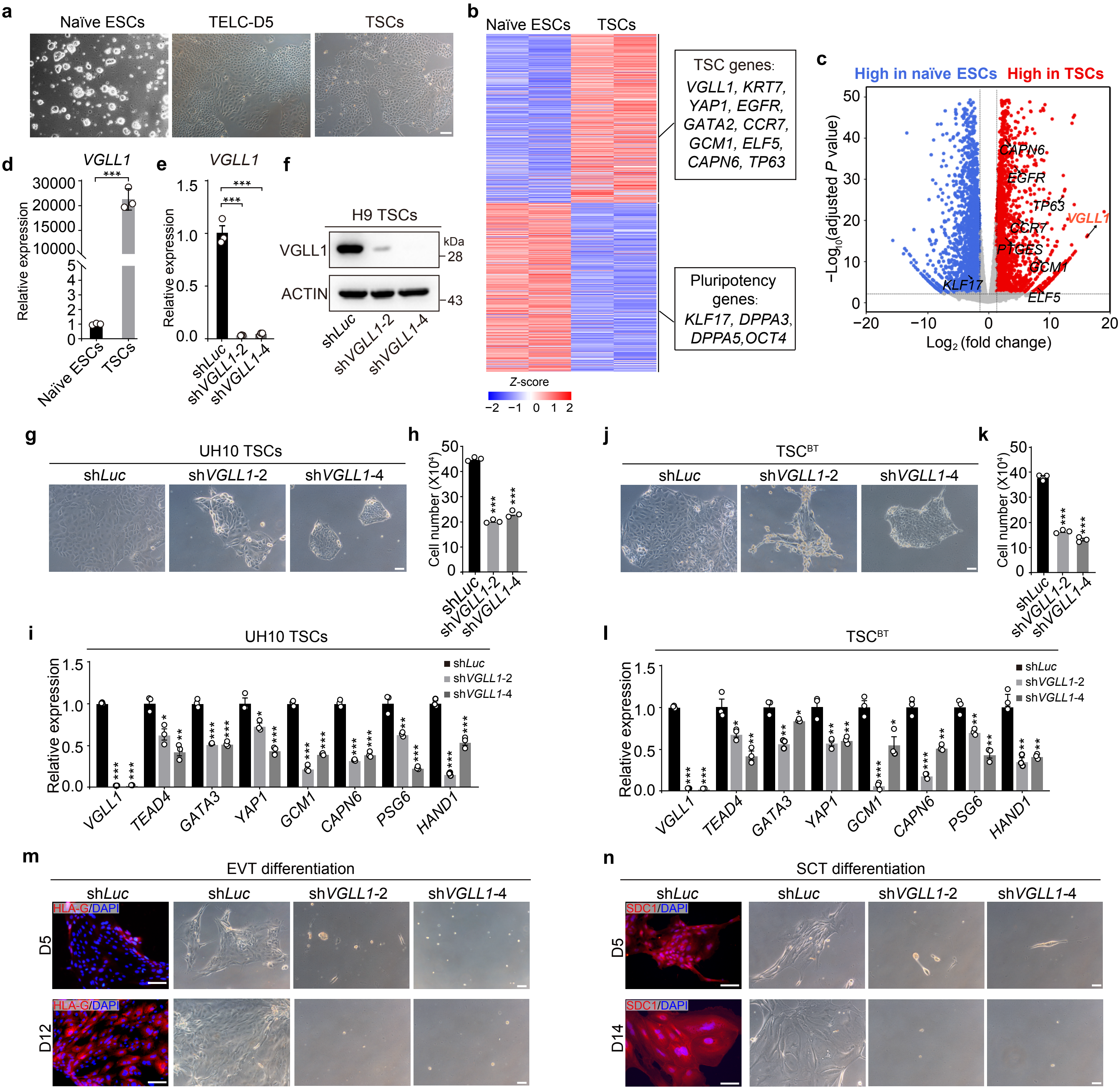

Supplementary Fig. 6

Supplementary Fig. 6. VGLL1 is required for maintaining the identity of human TSCs derived from PSCs or embryos.

a. Phase contrast images of 4CL H9 ESCs and TELC-D5 cells derived from them, and TSCs derived from TELC-D5 cells. Scale bar, 40 μm. Representative of three independent experiments.

b. Heatmap showing the expression of pluripotency- and TSC-related genes for the indicated conditions. Example genes are shown for each cluster in the boxes. n=2 biological replicates.

c. Volcano plot showing DEGs between 4CL H9 ESCs and TSCs derived from them. DEGs higher in TSCs (Log_2 (fold change) > 1) are shown in red. *P* value was calculated using the Wald test and adjusted for multiple testing using the Benjamini-Hochberg correction.

d. RT-qPCR showing *VGLL1* expression in 4CL H9 ESCs and TSCs derived from them. Data are presented as the mean ± SEM, n=3 biological replicates. *P* value was calculated using a two-tailed unpaired Student's *t*-test, ***$P < 0.001$.

e. RT-qPCR showing the *VGLL1* knockdown efficiency for sh*VGLL1*-2 and sh*VGLL1*-4 compared to the sh*Luc* control in 4CL H9 ESC-derived TSCs. Data are presented as the mean ± SEM, n=3 biological replicates. *P* value was calculated using a two-tailed unpaired Student's *t*-test, ***$P < 0.001$.

f. Western blotting of the indicated proteins for sh*VGLL1*-2 and sh*VGLL1*-4 compared to the sh*Luc* control in 4CL H9 ESC-derived TSCs. Representative of three independent experiments.

g. Representative phase contrast images of 4CL UH10 iPSC-derived TSCs transduced with sh*Luc*, sh*VGLL1*-2 or sh*VGLL1*-4. Scale bar, 100 μm. Representative of three independent experiments.

h. Analysis of cell numbers for 4CL UH10 iPSC-derived TSCs transduced with sh*Luc*, sh*VGLL1*-2 or sh*VGLL1*-4. Data are presented as the mean ± SEM. n=3 biological replicates. *P* value was calculated using a two-tailed unpaired Student's *t*-test, ***$P < 0.001$.

i. RT-qPCR showing the expression of TSC-related genes for 4CL UH10 iPSC-derived TSCs transduced with sh*Luc*, sh*VGLL1*-2 or sh*VGLL1*-4. Data are presented as the mean ± SEM.

n=3 biological replicates. *P* value was calculated using a two-tailed unpaired Student's *t*-test, ****P* < 0.001, ***P* < 0.01, **P* < 0.05.

j. Representative phase contrast images of TSC^{BT} transduced with sh*Luc*, sh*VGLL1*-2 or sh*VGLL1*-4. Scale bar, 100 µm. Representative of three independent experiments.

k. Analysis of cell numbers for TSC^{BT} transduced with sh*Luc*, sh*VGLL1*-2 or sh*VGLL1*-4. Data are presented as the mean ± SEM. n=3 biological replicates. *P* value was calculated using a two-tailed unpaired Student's *t*-test, ****P* < 0.001.

l. RT-qPCR showing the expression of TSC-related genes for TSC^{BT} transduced with sh*Luc*, sh*VGLL1*-2 or sh*VGLL1*-4. Data are presented as the mean ± SEM. n=3 biological replicates. *P* value was calculated using a two-tailed unpaired Student's *t*-test, ****P* < 0.001, ***P* < 0.01, **P* < 0.05.

m. Representative phase contrast images of 4CL H9 ESC-derived TSCs transduced with sh*Luc*, sh*VGLL1*-2 or sh*VGLL1*-4 at day 5 (upper panels) and day 12 (lower panels) of EVT differentiation. Immunostaining images of HLA-G for sh*Luc* control, nuclei were counterstained with DAPI (left panels). Scale bar, 100 µm. Representative of three independent experiments.

n. Representative phase contrast images of 4CL H9 ESC-derived TSCs transduced with sh*Luc*, sh*VGLL1*-2 or sh*VGLL1*-4 at day 5 (upper panels) and day 14 (lower panels) of SCT differentiation. Immunostaining images of SDC1 for sh*Luc* control, nuclei were counterstained with DAPI (left panels). Scale bar, 100 µm. Representative of three independent experiments.

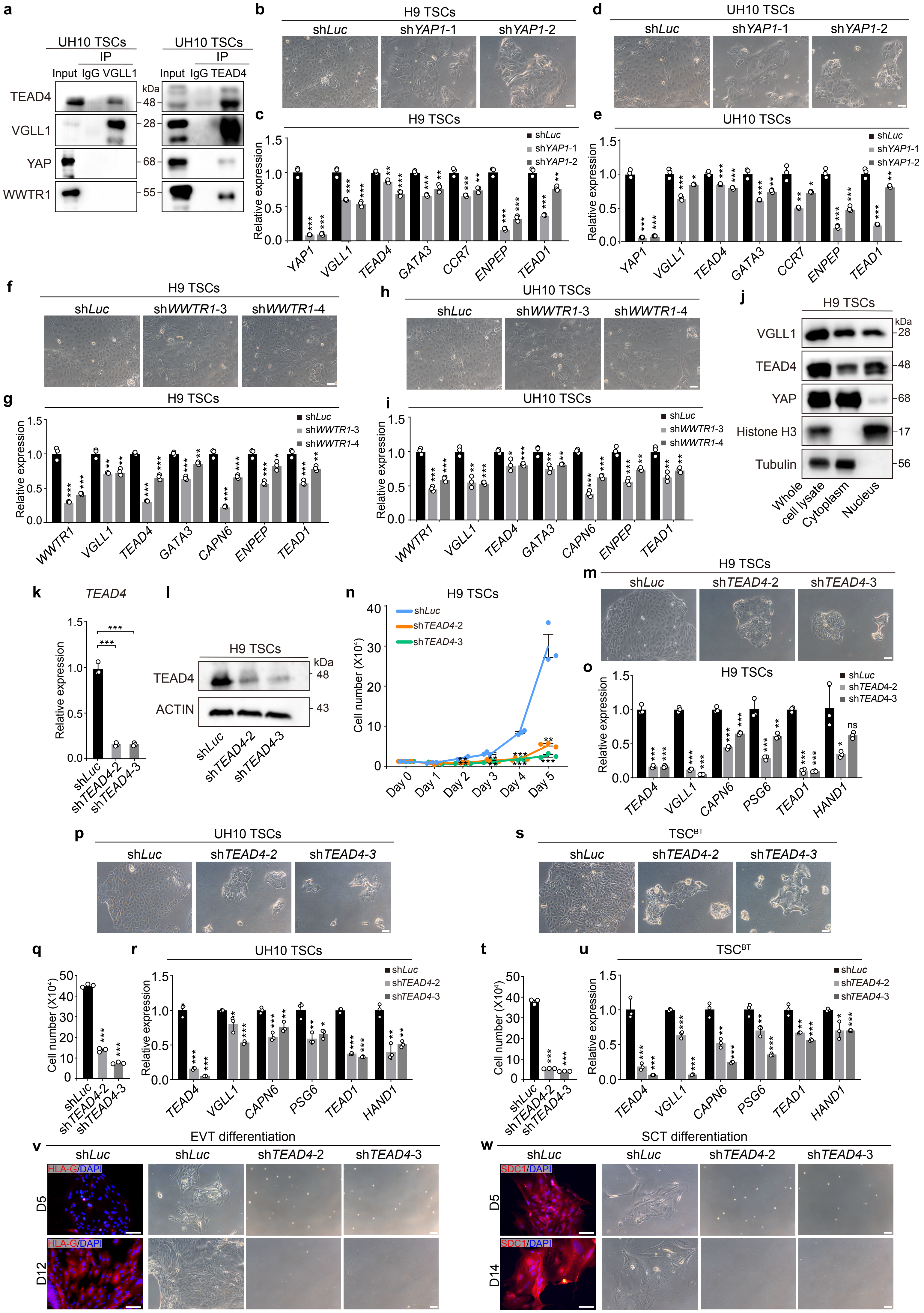

Supplementary Fig. 7

Supplementary Fig. 7. Role of YAP1, WWTR1 and TEAD4 in maintaining human TSC identity.

a. Immunoprecipitation using 4CL UH10 iPSC-derived TSC lysates with anti-VGLL1 (left panel) or anti-TEAD4 (right panel) and subsequent Western blotting with anti-TEAD4, anti-VGLL1, anti-YAP and anti-WWTR1. Representative of three independent experiments.

b. Phase contrast images of 4CL H9 ESC-derived TSCs transduced with sh*Luc*, sh*YAP1*-1 or sh*YAP1*-2. Scale bar, 100 µm. Representative of three independent experiments.

c. RT-qPCR showing the expression of TSC-related genes in 4CL H9 ESC-derived TSCs transduced with sh*Luc*, sh*YAP1*-1or sh*YAP1*-2. Data are presented as the mean ± SEM. n=3 biological replicates. *P* value was calculated using a two-tailed unpaired Student's *t*-test, $***P < 0.001$, $**P < 0.01$.

d. Phase contrast images of 4CL UH10 iPSC-derived TSCs transduced with sh*Luc*, sh*YAP1*-1or sh*YAP1*-2. Scale bar, 100 µm. Representative of three independent experiments.

e. RT-qPCR showing the expression of TSC-related genes for 4CL UH10 iPSC-derived TSCs transduced with sh*Luc*, sh*YAP1*-1or sh*YAP1*-2. Data are presented as the mean ± SEM. n=3 biological replicates. *P* value was calculated using a two-tailed unpaired Student's *t*-test, $***P < 0.001$, $**P < 0.01$, $*P < 0.05$.

f. Phase contrast images of 4CL H9 ESC-derived TSCs transduced with sh*Luc*, sh*WWTR1*-3 or sh*WWTR1*-4. Scale bar, 100 µm. Representative of three independent experiments.

g. RT-qPCR showing the expression of TSC-related genes for 4CL H9 ESC-derived TSCs transduced with sh*Luc*, sh*WWTR1*-3 or sh*WWTR1*-4. Data are presented as the mean ± SEM. n=3 biological replicates. *P* value was calculated using a two-tailed unpaired Student's *t*-test, $***P < 0.001$, $**P < 0.01$, $*P < 0.05$.

h. Phase contrast images of 4CL UH10 iPSC-derived TSCs transduced with sh*Luc*, sh*WWTR1*-3 or sh*WWTR1*-4. Scale bar, 100 µm. Representative of three independent experiments.

i. RT-qPCR showing the expression of TSC-related genes for 4CL UH10 iPSC-derived TSCs transduced with sh*Luc*, sh*WWTR1*-3 or sh*WWTR1*-4. Data are presented as the mean ±

SEM. n=3 biological replicates. *P* value was calculated using a two-tailed unpaired Student's *t*-test, $^{***}P < 0.001$, $^{**}P < 0.01$, $^{*}P < 0.05$.

j. Fractionation Western blotting analysis for the indicated proteins in 4CL H9 ESC-derived TSCs.

k. RT-qPCR showing the *TEAD4* knockdown efficiency for sh*TEAD4*-2 and sh*TEAD4*-3 compared to the sh*Luc* control in 4CL H9 ESC-derived TSCs. Data are presented as the mean ± SEM, n=3 biological replicates. *P* value was calculated using a two-tailed unpaired Student's *t*-test, $^{***}P < 0.001$.

l. Western blotting for the indicated proteins in 4CL H9 ESC-derived TSCs transduced with sh*TEAD4*-2 or sh*TEAD4*-3 compared to sh*Luc* control. Representative of three independent experiments.

m. Phase contrast images of 4CL H9 ESC-derived TSCs transduced with sh*Luc*, sh*TEAD4*-2 or sh*TEAD4*-3. Scale bar, 100 μm. Representative of three independent experiments.

n. Analysis of cell numbers for 4CL H9 ESC-derived TSCs transduced with sh*Luc*, sh*TEAD4*-2 or sh*TEAD4*-3. Data are presented as the mean ± SEM, n=3 biological replicates. *P* value was calculated using a two-tailed unpaired Student's *t*-test, $^{***}P < 0.001$, $^{**}P < 0.01$.

o. RT-qPCR showing the expression of TSC-related genes in 4CL H9 ESC-derived TSCs transduced with sh*Luc*, sh*TEAD4*-2 or sh*TEAD4*-3. Data are presented as the mean ± SEM. n=3 biological replicates. *P* value was calculated using a two-tailed unpaired Student's *t*-test, $^{***}P < 0.001$, $^{**}P < 0.01$, $^{*}P < 0.05$. ns: not significant.

p. Phase contrast images of 4CL UH10 iPSC-derived TSCs transduced with sh*Luc*, sh*TEAD4*-2 or sh*TEAD4*-3. Scale bar, 100 μm. Representative of three independent experiments.

q. Analysis of cell numbers for 4CL UH10 iPSC-derived TSCs transduced with sh*Luc*, sh*TEAD4*-2 or sh*TEAD4*-3. Data are presented as the mean ± SEM, n=3 biological replicates. *P* value was calculated using a two-tailed unpaired Student's *t*-test, $^{***}P < 0.001$.

r. RT-qPCR showing the expression of TSC-related genes for 4CL UH10 ESC-derived TSCs transduced with sh*Luc*, sh*TEAD4*-2 or sh*TEAD4*-3. Data are presented as the mean ± SEM. n=3 biological replicates. *P* value was calculated using a two-tailed unpaired Student's *t*-test, $^{***}P < 0.001$, $^{**}P < 0.01$, $^{*}P < 0.05$.

s. Representative phase contrast images of TSC^{BT} transduced with sh*Luc*, sh*TEAD4*-2 or sh*TEAD4*-3. Scale bar, 100 µm. Representative of three independent experiments.

t. Analysis of cell numbers for TSC^{BT} transduced with sh*Luc*, sh*TEAD4*-2 or sh*TEAD4*-3. Data are presented as the mean ± SEM. n=3 biological replicates. *P* value was calculated using a two-tailed unpaired Student's *t*-test, $***P < 0.001$.

u. RT-qPCR showing the expression of TSC-related genes for TSC^{BT} transduced with sh*Luc*, sh*TEAD4*-2 or sh*TEAD4*-3. Data are presented as the mean ± SEM. n=3 biological replicates. *P* value was calculated using a two-tailed unpaired Student's *t*-test, $***P < 0.001$, $**P < 0.01$, $*P < 0.05$.

v. Representative phase contrast images of 4CL H9 ESC-derived TSCs transduced with sh*Luc*, sh*TEAD4*-2 or sh*TEAD4*-3 at day 5 (upper panel) and day 12 (lower panel) of EVT differentiation. Immunostaining images of HLA-G for sh*Luc* control, nuclei were counterstained with DAPI (left panels). Scale bar, 100 µm. Representative of three independent experiments.

w. Representative phase contrast images of 4CL H9 ESC-derived TSCs transduced with sh*Luc*, sh*TEAD4*-2 or sh*TEAD4*-3 at day 5 (upper panel) and day 14 (lower panel) of SCT differentiation. Immunostaining images of SDC1 for sh*Luc* control, nuclei were counterstained with DAPI (left panels). Scale bar, 100 µm. Representative of three independent experiments.

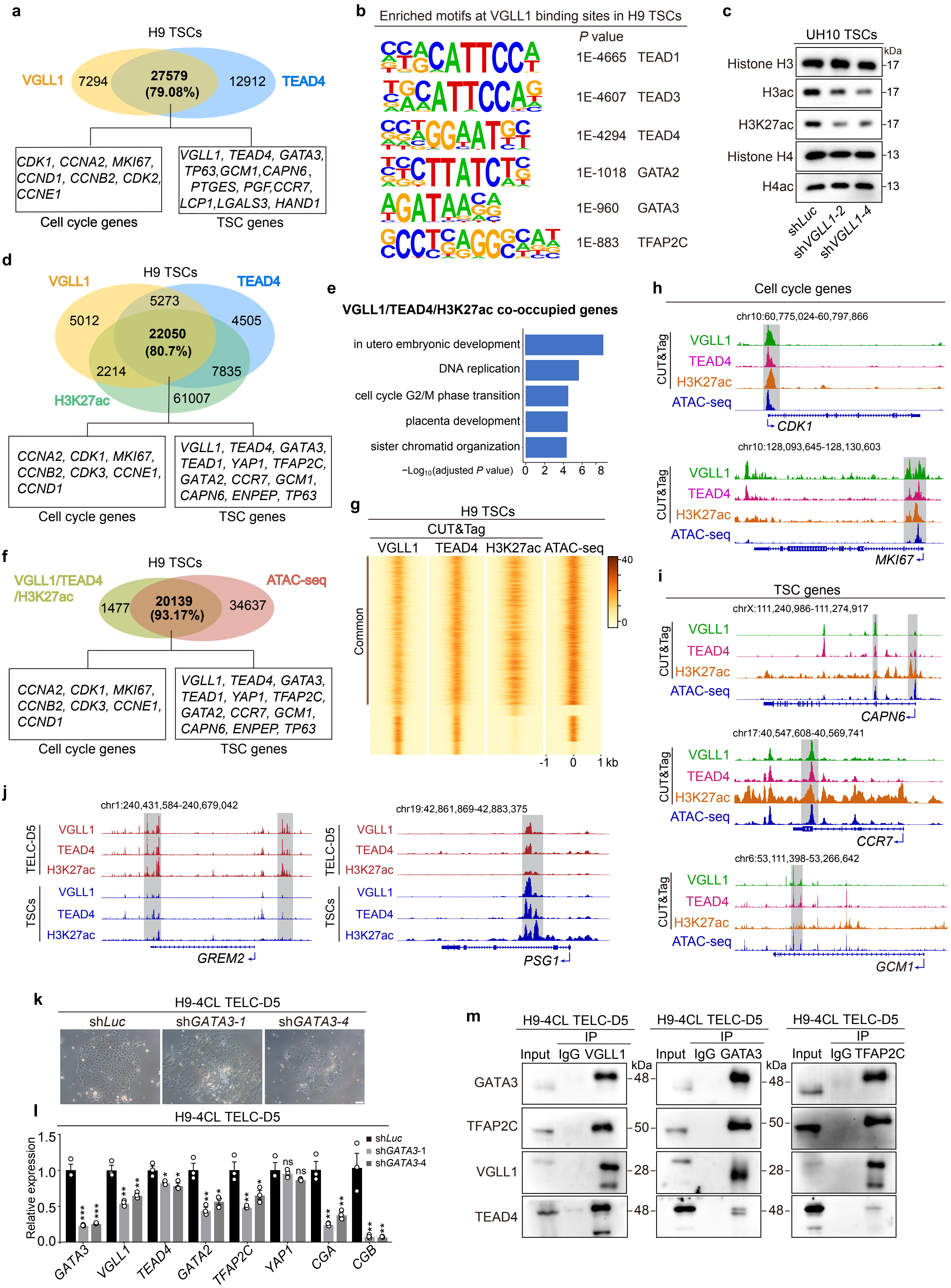

Supplementary Fig. 8

Supplementary Fig. 8. VGLL1/TEAD4/H3K27ac enrichment correlates with chromatin accessibility in human TSCs.

- **a.** Venn diagram showing the overlap between VGLL1 and TEAD4 co-bound sites in CUT&Tag of 4CL H9 ESC-derived TSCs. Representative cell cycle- and TSC-related genes corresponding to the overlapping regions are shown.
- **b.** Motif analysis using HOMER showing the significantly enriched DNA-binding motifs at VGLL1 bound sites in 4CL H9 ESC-derived TSCs.
- **c.** Western blotting of the indicated histone marks for sh*VGLL1*-2 and sh*VGLL1*-4 compared to the sh*Luc* control in 4CL UH10 iPSC-derived TSCs. Representative of three independent experiments.
- **d.** Venn diagram showing the overlap between VGLL1, TEAD4, and H3K27ac occupied sites in CUT&Tag of 4CL H9 ESC-derived TSCs. Representative cell cycle- and TSC-related genes corresponding to the overlapping regions are shown.
- **e.** Enriched GO terms for VGLL1, TEAD4 and H3K27ac co-occupied sites in CUT&Tag of 4CL H9 ESC-derived TSCs in panel d. *P* value was calculated using a hypergeometric test and adjusted for multiple testing using the Benjamini-Hochberg correction.
- **f.** Venn diagram showing the overlap between VGLL1, TEAD4 and H3K27ac co-occupied sites in CUT&Tag with chromatin accessibility peaks (ATAC-seq) in 4CL H9 ESC-derived TSCs. Representative cell cycle- and TSC-related genes corresponding to the overlapping regions are shown.
- **g.** Co-occupancy analysis by signal density pileups of VGLL1, TEAD4, and H3K27ac genomic enrichment peaks (CUT&Tag) and chromatin accessibility peaks (ATAC-seq) in 4CL H9 ESC-derived TSCs.
- **h.** **h** and **i**. Genome browser tracks showing VGLL1, TEAD4 and H3K27ac genomic enrichment peaks (CUT&Tag) and chromatin accessibility peaks (ATAC-seq) for representative cell cycle (*CDK1*, *MKI67*) (**h**) and TSC-related (*CAPN6*, *CCR7* and *GCM1*) (**i**) gene loci in 4CL H9 ESC-derived TSCs.
- **j.** Genome browser tracks showing VGLL1, TEAD4 and H3K27ac genomic enrichment peaks (CUT&Tag) in 4CL H9 ESC-derived TELC-D5 cells and TSCs for representative gene loci from group 2 (left panel) and group 3 (right panel).

k. Phase contrast images of 4CL H9 ESCs transduced with sh*Luc*, sh*GATA3*-1, or sh*GATA3*-4 at day 5 of TELC induction. Scale bar, 100 μm. Representative of three independent experiments.

l. RT-qPCR showing the expression of TE genes for 4CL H9 ESCs transduced with sh*Luc*, sh*GATA3*-1 or sh*GATA3*-4 at day 5 of TELC differentiation. Data are presented as the mean ± SEM. n=3 biological replicates. *P* value was calculated using a two-tailed unpaired Student's *t*-test, ***$P < 0.001$, **$P < 0.01$, *$P < 0.05$, ns: not significant.

m. Immunoprecipitation using 4CL H9 ESC-derived TELC-D5 cell lysates with anti-VGLL1 (left panel), anti-GATA3 (middle panel) and anti-TFAP2C (right panel) and subsequent Western blotting with anti-GATA3, anti-TFAP2C, anti-VGLL1 and anti-TEAD4. Representative of three independent experiments.

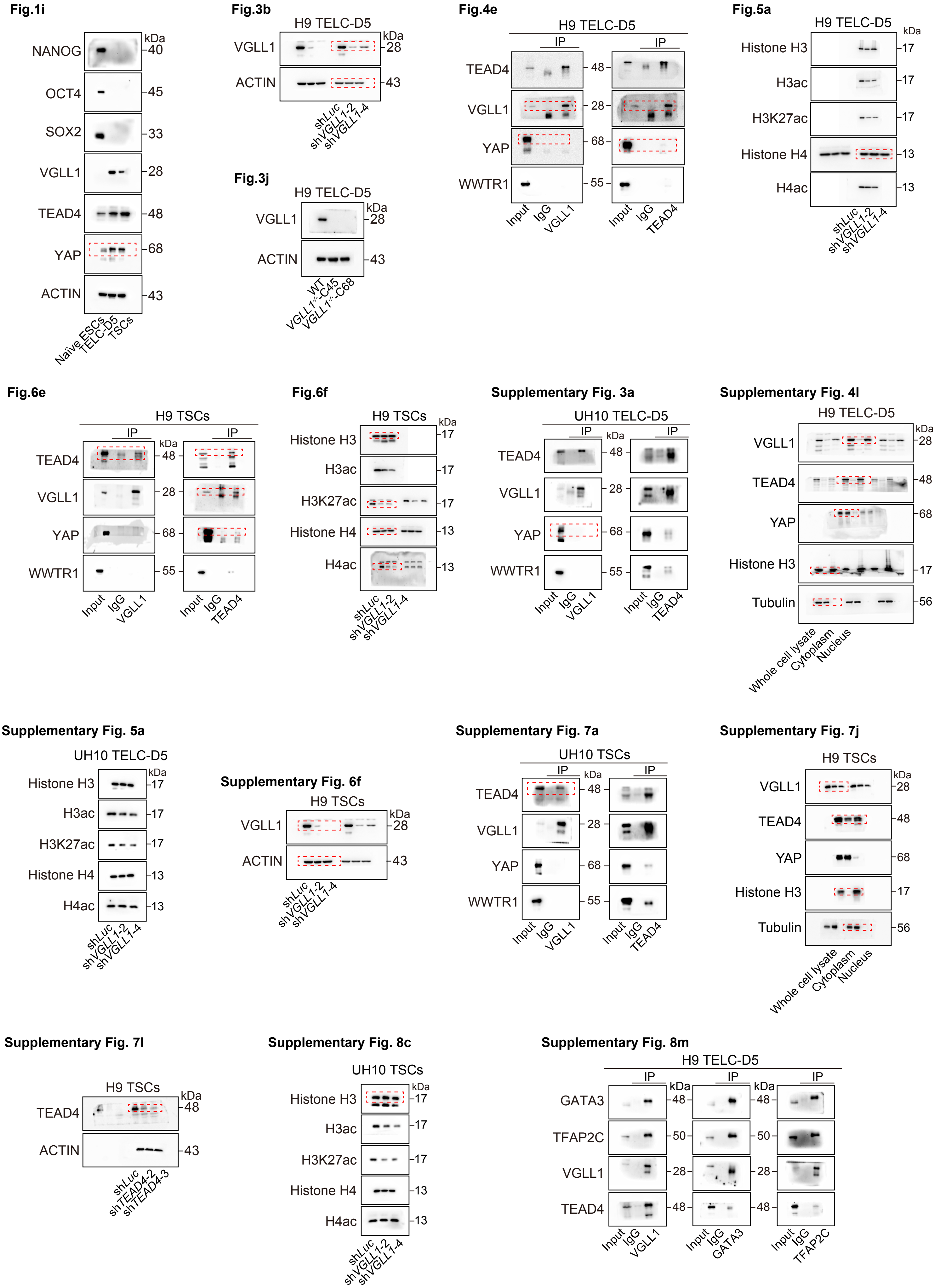

Supplementary Fig. 9. Uncropped scans of Western blots presented in all figures.

Supplementary tables

Supplementary Table 1: shRNAs used in this study.

shRNA	Sense	Antisense
sh*VGLL1*-2	GCAAGACATCCCTGCATATCTCGAGATATGCAGGGATGTCTTGC	GCAAGACATCCCTGCATATCTCGAGATATGCAGGGATGTCTTGC
sh*VGLL1*-4	GCGTAGTGGATGAACACTTCTCGAGAAGTGTTCATCCACTACGC	GCGTAGTGGATGAACACTTCTCGAGAAGTGTTCATCCACTACGC
sh*TEAD4*-2	ATGATCAACTTCATCCACAAGCTCGAGCTTGTGGATGAAGTTGATCAT	ATGATCAACTTCATCCACAAGCTCGAGCTTGTGGATGAAGTTGATCAT
sh*TEAD4*-3	GATCAACTTCATCCACAAGCTCTCGAGAGCTTGTGGATGAAGTTGATC	GATCAACTTCATCCACAAGCTCTCGAGAGCTTGTGGATGAAGTTGATC
sh*YAP1*-1	CTGGTCAGAGATACTTCTTAACTCGAGTTAAGAAGTATCTCTGACC	CTGGTCAGAGATACTTCTTAACTCGAGTTAAGAAGTATCTCTGACC
sh*YAP1*-2	AAGCTTTGAGTTCTGACATCCCTCGAGGGATGTCAGAACTCAAAGC	AAGCTTTGAGTTCTGACATCCCTCGAGGGATGTCAGAACTCAAAGC
sh*WWTR1*-3	GCCCTTTCTAACCTGGCTGTACTCGAGTACAGCCAGGTTAGAAAGGGC	GCCCTTTCTAACCTGGCTGTACTCGAGTACAGCCAGGTTAGAAAGGGC
sh*WWTR1*-4	CCTGCCGGAGTCTTTCTTTAACTCGAGTTAAAGAAAGACTCCGGCAGG	CCTGCCGGAGTCTTTCTTTAACTCGAGTTAAAGAAAGACTCCGGCAGG
sh*GATA3*-1	CATCCAGACCAGAAACCGAAACTCGAGTTTCGGTTTCTGGTCTGGATG	CATCCAGACCAGAAACCGAAACTCGAGTTTCGGTTTCTGGTCTGGATG
sh*GATA3*-4	AGCCTAAACGCGATGGATATACTCGAGTATATCCATCGCGTTTAGGCT	AGCCTAAACGCGATGGATATACTCGAGTATATCCATCGCGTTTAGGCT
sh*TP53*-1	CGGCGCACAGAGGAAGAGAATCTCGAGATTCTCTTCCTCTGTGCGCCG	CGGCGCACAGAGGAAGAGAATCTCGAGATTCTCTTCCTCTGTGCGCCG
sh*TP53*-2	GTCCAGATGAAGCTCCCAGAACTCGAGTTCTGGGAGCTTCATCTGGAC	GTCCAGATGAAGCTCCCAGAACTCGAGTTCTGGGAGCTTCATCTGGAC

Supplementary Table 2: sgRNAs used in this study.

sgRNA	Sense	Antisense
VGLL1-sgRNA1	GGTGTGTCCTTTTCACCTAC	GTAGGTGAAAAGGACACACC

TEAD4-sgRNA2	TCGTCCGACAGGATGATTTT	AAAATCATCCTGTCGGACGA

Supplementary Table 3: Primers used for genomic PCR in this study.

Primer	Sense	Antisense
VGLL1	CCCCAGGAGGCAAGTTACTC	CCCCACATGCTCACCGTTTT
TEAD4	CTGAGCAACTGATTCGGGCT	AGTGTCCACCCGACAAAGC

Supplementary Table 4: Primers used for RT-qPCR in this study.

Primer	Sense	Antisense
VGLL1	CTCCCGGCTCAGTTCACTATAA	CCCAGTGGTTTGGTGGTGTA
GATA3	GCCCCTCATTAAGCCCAAG	TTGTGGTGGTCTGACAGTTCG
CGA	TGCCCAGAATGCACGCTAC	TTGGACCTTAGTGGAGTGGGA
CGB	CAGCATCCTATCACCTCCTGGT	CTGGAACATCTCCATCCTTGGT
TEAD4	CAGGTGGTGGAGAAAGTTGAGA	GTGCTTGAGCTTGTGGATGAAG
GATA2	ACTGACGGAGAGCATGAAGAT	CCGGCACATAGGAGGGGTA
TFAP2C	CTGTTGCTGCACGATCAGACA	CTCAGTGGGGTTCATTACGGC
TFAP2A	GCTGCCTCACCAGCTGTCGG	GAGACGGCATTGCTGTTGGAC
YAP1	TAGCCCTGCGTAGCCAGTTA	TCATGCTTAGTCCACTGTCTGT
HAVCR1	CAGGTCCATCTGTCACACTAC	TAGCTTATAGCGTGTGTCC
TEAD1	GAAACTGGACACAAGTCAG	CTAGAGTGCAGAAGATTCAC
ENPEP	GATGAGAGCGGACAGTGG	AGGTGCAGCCACAGGTAC
HAND1	CCAAGGATGCACAGTCTGG	CGGTGCGTCCTTTAATCCT
NR2F2	ATGTTACTGTCCGGCAGCAG	GTAAAAGTTCATTTGACAGTC
KLF5	TCAGACAGCAGCAATGGACACTC	GTGGCCTGTTGTGGAAGAAACTG
WWTR1	ATGATGTAGAGTCTGCTCTG	AGGAGGGAGCACGAGTCATG
TP53	GATGAAGCTCCCAGAATGCC	TGCAAGTCACAGACTTGGC
CCR7	ACAGCCTTCCTGTGTGGTTTTA	CCAGCACGCTTTTCATTGGTT
PSG6	CCTGCTCAGGAAGTCTCTGG	CGGAAACTTTGGGTGGCTTG
GCM1	TTCCCGGTCACCAACTTCTG	GTAAACTCCCCTGACTTTGTGTT
CAPN6	ATGATCCCCTGATGAACCGC	CTTCCCATTCGGCGGTAAGT

Supplementary Table 5: List of antibodies used in this study.

Antibody	Company	Cat. No.	Usage	Dilution ratio (WB)
VGLL1 antibody	Invitrogen	PA5-59968	Co-IP, WB, IF, CUT&Tag	1:1000
Anti-VGLL1	Sigma-Aldrich	HPA064616	WB	1:1000
TEAD4 antibody	Novus	NBP1-32765	Co-IP, WB, IF, CUT&Tag	1:1000

Anti-TEAD4 antibody	Abcam	ab58310	WB	1:1000
YAP antibody	Santa Cruz Biotechnology	sc-101199	WB, CUT&Tag	1:1000
WWTR1 antibody	Proteintech	CL488-66500	WB	1:1000
beta Actin antibody	Santa Cruz Biotechnology	sc-47778	WB	1:1000
Oct3/4 antibody	Santa Cruz Biotechnology	sc-5279	WB	1:1000
SOX2 antibody	R&D Systems	AF2018	WB	1:1000
Anti-Nanog	Abcam	ab21624	WB, IF	1:1000
Anti-alpha Tubulin	Abcam	ab7291	WB	1:1000
Anti-Histone H3 antibody	Abcam	ab1791	WB	1:5000
Anti-Histone H4 antibody	Abcam	ab10158	WB	1:5000
Anti-acetyl-Histone H3 antibody	Sigma-Aldrich	06-599	WB	1:2000
Histone H4ac antibody	Active Motif	39926	WB	1:1000
Anti-Histone H3 (acetyl K27) antibody	Abcam	ab4729	WB, CUT&Tag	1:2000
AP-2γ antibody	Santa Cruz Biotechnology	sc-12762	Co-IP, WB, IF, CUT&Tag	1:1000
GATA3 antibody	Santa Cruz Biotechnology	sc-268	Co-IP, WB, IF, CUT&Tag	1:1000
KLF17 antibody	Sigma-Aldrich	HPA024629	IF	
Anti-HLA G antibody	Abcam	ab283260	IF	1:100
Anti-SDC1 antibody	Abcam	ab39969	IF	1:200
IgG (rabbit)	Beyotime	A7058	Co-IP	
IgG (mouse)	Beyotime	A7050	Co-IP	
Alexa Fluor 488-AffiniPure Donkey Anti-	Jackson ImmunoResearch	715-545-150	IF	1:500

Mouse IgG (H+L)				
Alexa Fluor 488-AffifiniPure Donkey Anti-Rabbit IgG (H+L)	Jackson ImmunoResearch	711-545-152	IF	1:500
Cy3-AffifiniPure Donkey Anti-Rabbit IgG (H+L)	Jackson ImmunoResearch	711-165-152	IF	1:500
Goat anti-Rabbit IgG (H+L) Secondary Antibody, HRP	Thermo Fisher Scientific	31460	WB	1:5000
Goat anti-Mouse IgG (H+L) Secondary Antibody, HRP	Thermo Fisher Scientific	31430	WB	1:5000
Dilution ratio of antibodies (except for HLA G and SDC1) used in immunofluorescence (IF)：1:400				
