# Supplementary material for: VGLL1 cooperates with TEAD4 to control human trophectoderm lineage specification
Source: Nat Commun. 2024 Jan 17;15:583. doi: 10.1038/s41467-024-44780-8 (PMC10794710; doi:10.1038/s41467-024-44780-8)
Supplement: Supplementary file 3 — Description of Additional Supplementary Files [file 41467_2024_44780_MOESM3_ESM.pdf]

Description of Additional Supplementary Files

File Name: Supplementary Data 1
Description: List of VGLL1 binding loci in TELC-D5 cells including annotation information. Related to Fig. 4a.
