# Supplementary material for: VGLL1 cooperates with TEAD4 to control human trophectoderm lineage specification
Source: Nat Commun. 2024 Jan 17;15:583. doi: 10.1038/s41467-024-44780-8 (PMC10794710; doi:10.1038/s41467-024-44780-8)
Supplement: Supplementary file 5 — Reporting Summary [file 41467_2024_44780_MOESM5_ESM.pdf]

nature portfolio

Corresponding author(s): Miguel A. Esteban
Md. Abdul Mazid
Wenjuan Li

Last updated by author(s): Dec 19, 2023

Reporting Summary

Nature Portfolio wishes to improve the reproducibility of the work that we publish. This form provides structure for consistency and transparency in reporting. For further information on Nature Portfolio policies, see our Editorial Policies and the Editorial Policy Checklist.

Statistics

For all statistical analyses, confirm that the following items are present in the figure legend, table legend, main text, or Methods section.

n/a	Confirmed	
☐	☒	The exact sample size (*n*) for each experimental group/condition, given as a discrete number and unit of measurement
☐	☒	A statement on whether measurements were taken from distinct samples or whether the same sample was measured repeatedly
☐	☒	The statistical test(s) used AND whether they are one- or two-sided<br>*Only common tests should be described solely by name; describe more complex techniques in the Methods section.*
☐	☒	A description of all covariates tested
☐	☒	A description of any assumptions or corrections, such as tests of normality and adjustment for multiple comparisons
☐	☒	A full description of the statistical parameters including central tendency (e.g. means) or other basic estimates (e.g. regression coefficient) AND variation (e.g. standard deviation) or associated estimates of uncertainty (e.g. confidence intervals)
☐	☒	For null hypothesis testing, the test statistic (e.g. F, t, r) with confidence intervals, effect sizes, degrees of freedom and P value noted<br>*Give P values as exact values whenever suitable.*
☒	☐	For Bayesian analysis, information on the choice of priors and Markov chain Monte Carlo settings
☒	☐	For hierarchical and complex designs, identification of the appropriate level for tests and full reporting of outcomes
☐	☒	Estimates of effect sizes (e.g. Cohen's *d*, Pearson's *r*), indicating how they were calculated

Our web collection on statistics for biologists contains articles on many of the points above.

Software and code

Policy information about availability of computer code

Data collection	ABI 7500 real-time PCR machine for RT-qPCR, FusionCapt Advance Solo 4.16.15 for Western blot, ZEN software v2.0. for immunofluorescence, DNBSEQ platform and Illumina NovaSeq 6000 platform for bulk RNA-seq, DIPSEQ T1 platform for scRNA-seq, Illumina NovaSeq 6000 platform for CUT&Tag and bulk ATAC-seq, results presented as mean ± SEM were collected and calculated using GraphPad Prism (v8).
Data analysis	Data were analysed using: fastp (v0.21.0), STAR (v2.7.3a), RSEM (v1.2.18), DESeq2 (v1.24), ClusterProfiler (v3.6.0), ggplot2 (v3.3.3), pheatmap (v1.0.12), EnhancedVolcano (v1.8.0), Bowtie2 (v2.2.5), Samtools (v1.10), Picard (v1.9.0), sambamba (v0.7.0), MACS2 (v2.1.0), ChIPseeker (v1.26.2), ChIPpeakAnno (v3.24.2), deeptools (v3.4.3), Seurat (v3.1.4) and Monocle 2 (v4.1.0).

For manuscripts utilizing custom algorithms or software that are central to the research but not yet described in published literature, software must be made available to editors and reviewers. We strongly encourage code deposition in a community repository (e.g. GitHub). See the Nature Portfolio guidelines for submitting code & software for further information.

Data

Policy information about availability of data

All manuscripts must include a data availability statement. This statement should provide the following information, where applicable:

- Accession codes, unique identifiers, or web links for publicly available datasets
- A description of any restrictions on data availability
- For clinical datasets or third party data, please ensure that the statement adheres to our policy

All bulk sequencing data (RNA-seq, ATAC-seq and CUT&Tag) reported in this paper are deposited in the Gene Expression Omnibus (GEO) database under the accession number GSE193621 [https://www.ncbi.nlm.nih.gov/geo/query/acc.cgi?acc=GSE193621]. Raw single cell sequencing data reported in this paper are deposited in the CNGB Nucleotide Sequence Archive under the accession number CNP0004944 [https://db.cngb.org/search/project/CNP0004944/]. Public scRNA-seq dataset of human early embryo development were downloaded from EBI ArrayExpress (E-MTAB-3929 [https://www.ebi.ac.uk/biostudies/arrayexpress/studies/E-MTAB-3929?query=E-MTAB-3929]), ENA database (PRJEB30442 [https://www.ebi.ac.uk/ena/browser/view/PRJEB30442]) and GEO database (GSE136447 [https://www.ncbi.nlm.nih.gov/geo/query/acc.cgi?acc=GSE136447]). scRNA-seq datasets of monkey and mouse early embryo development were downloaded from GEO database (GSE7476748 [https://www.ncbi.nlm.nih.gov/geo/query/acc.cgi?acc=GSE74767], GSE8489249 [https://www.ncbi.nlm.nih.gov/geo/query/acc.cgi?acc=GSE84892]). Bulk RNA-seq and scRNA-seq dataset of 4CL H9 naïve ESCs were downloaded from CNGBdb (CNP0001454 [https://db.cngb.org/search/project/CNP0001454/]).

Research involving human participants, their data, or biological material

Policy information about studies with human participants or human data. See also policy information about sex, gender (identity/presentation), and sexual orientation and race, ethnicity and racism.

Reporting on sex and gender	Sex or gender was not considered in the study design. However, the results of this study were validated in human H9 ESCs and human UH10 iPSCs, which are female and male cell line respectively. Thus, the conclusion of this study were not only apply to one sex.
Reporting on race, ethnicity, or other socially relevant groupings	Not applicable.
Population characteristics	Not applicable.
Recruitment	Not applicable.
Ethics oversight	The use of human H9 ESCs (WiCell, WA09), UH10 iPSCs and TSCBT in this study is compliant with the 'Guidance of the Ministry of Science and Technology for the Review and Approval of Human Genetic Resources' and was approved by the 'Life Science and Medical Ethics Committee' of the Guangzhou Institutes of Biomedicine and Health under license number GIBH-LMEC2023-001-01(AL).

Note that full information on the approval of the study protocol must also be provided in the manuscript.

Field-specific reporting

Please select the one below that is the best fit for your research. If you are not sure, read the appropriate sections before making your selection.

☒ Life sciences ☐ Behavioural & social sciences ☐ Ecological, evolutionary & environmental sciences

For a reference copy of the document with all sections, see nature.com/documents/nr-reporting-summary-flat.pdf

Life sciences study design

All studies must disclose on these points even when the disclosure is negative.

Sample size	The sample size was estimated from the preliminary experiments or from our previously published studies. No statistical method was applied to predetermine sample size. RT-qPCR were conducted with at least three independent experiments except otherwise stated. Bulk RNA-seq, scRNA-seq, ATAC-seq and CUT&Tag were conducted with two or three independent samples. Please refer to figure legends and methods for details. All replications were successful.
Data exclusions	No data were excluded.
Replication	Each experiment was reproduced in at least three biological replicates unless otherwise stated. Please refer to figure legends and methods for details. All replications were successful.
Randomization	No randomization methods were utilized as all samples are randomly collected. Sample collections were performed with controls.
Blinding	No blinding was used. Blinding was not used because all analysis was performed on all relevant samples in reproducible ways, so no bias could have been removed by blinding. Besides, most of the experiments were performed by at least two independent researchers.

Reporting for specific materials, systems and methods

We require information from authors about some types of materials, experimental systems and methods used in many studies. Here, indicate whether each material, system or method listed is relevant to your study. If you are not sure if a list item applies to your research, read the appropriate section before selecting a response.

Materials & experimental systems

n/a	Involved in the study
☐	☒ Antibodies
☐	☒ Eukaryotic cell lines
☒	☐ Palaeontology and archaeology
☒	☐ Animals and other organisms
☒	☐ Clinical data
☒	☐ Dual use research of concern
☒	☐ Plants

Methods

n/a	Involved in the study
☐	☒ ChIP-seq
☒	☐ Flow cytometry
☒	☐ MRI-based neuroimaging

Antibodies

Antibodies used

Antibodies used:

For immunostaining:
Antibody, Company, Catalogue Number, Dilution
Anti-Nanog antibody, Abcam, Cat# ab21624, 1:400
KLF17 antibody, Sigma-Aldrich, Cat# HPA024629, 1:400
AP-2γ antibody, Santa Cruz Biotechnology, Cat# sc-12762, 1:400
TEAD4 antibody, Novus, Cat# NBP1-32765, 1:400
GATA3 antibody, Santa Cruz Biotechnology, Cat# sc-268, 1:400
VGLL1 antibody, Invitrogen, Cat# PA5-59968, 1:400
Anti-HlA G antibody, Abcam, Cat# ab283260, 1:100
Anti-SDC1 antibody, Abcam, Cat# ab39969, 1:200
Alexa Fluor 488-AffifiniPure Donkey Anti-Mouse IgG (H+L), Jackson ImmunoResearch, Cat# 715-545-150, 1:500
Alexa Fluor 488-AffifiniPure Donkey Anti-Rabbit IgG (H+L) , Jackson ImmunoResearch, Cat# 711-545-152, 1:500
Cy3-AffifiniPure Donkey Anti-Rabbit IgG (H+L), Jackson ImmunoResearch, Cat# 711-165-152, 1:500

For Western blotting:
Antibody, Company, Catalogue Number, Dilution
Anti-Nanog antibody, Abcam, Cat# ab21624, 1:1000
Oct3/4 antibody, Santa Cruz Biotechnology, Cat# sc-5279, 1:1000
SOX2 antibody, R&D Systems, Cat# AF2018, 1:1000
Anti-alpha Tubulin antibody, Abcam, Cat# ab7291, 1:1000
beta Actin antibody, Santa Cruz Biotechnology, Cat# sc-47778, 1:1000
VGLL1 antibody, Invitrogen, Cat# PA5-59968, 1:1000
Anti-VGLL1 antibody, Sigma-Aldrich, HPA064616, 1:1000
Anti-TEAD4 antibody, Abcam, ab58310, 1:1000
TEAD4 antibody, Novus, Cat# NBP1-32765, 1:1000
YAP antibody, Santa Cruz Biotechnology, Cat# sc-101199, 1:1000
GATA3 antibody, Santa Cruz Biotechnology, Cat# sc-268, 1:1000
AP-2γ antibody, Santa Cruz Biotechnology, Cat# sc-12762, 1:1000
WWTR1 antibody, Proteintech, Cat# CL488-66500, 1:1000
Anti-Histone H3 antibody, abcam, Cat# ab1791, 1:5000
Anti-Histone H4 antibody, abcam, Cat# ab10158, 1:5000
Anti-acetyl-Histone H3 antibody, Sigma-Aldrich, Cat# 06-599, 1:2000
Histone H4ac antibody, Active Motif, Cat# 39926, 1:1000
Anti-Histone H3 (acetyl K27) antibody, abcam, Cat# ab4729, 1:2000
Goat anti-Rabbit IgG (H+L) Secondary Antibody, HRP, Thermo Fisher Scientific, Cat# 31460, 1:5000
Goat anti-Mouse IgG (H+L) Secondary Antibody, HRP, Thermo Fisher Scientific, Cat# 31430, 1:5000

For Co-Immunoprecipitation:
Antibody, Company, Catalogue Number
VGLL1 antibody, Invitrogen, Cat# PA5-59968
TEAD4 antibody, Novus, Cat# NBP1-32765
GATA3 antibody, Santa Cruz Biotechnology, Cat# sc-268
AP-2γ antibody, Santa Cruz Biotechnology, Cat# sc-12762
IgG (rabbit), Beyotime, Cat# A7058

IgG (mouse), Beyotime, Cat# A7050

Details of all antibodies used in this study were provided in Supplementary Table 5 with catalog number, and commercial sources supplied.

Validation

Antibodies obtained from the commercial source were validated by the suppliers, detailed validation analysis relevant literatures are provided on the company website for the products used in this study. Some antibodies were validated in a previously published study as indicated in methods or relevant literature was cited.
1. VGLL1 (PA5-59968) https://www.thermofisher.com/antibody/product/VGLL1-Antibody-Polyclonal/PA5-59968
2. Anti-VGLL1 (HPA064616) https://www.sigmaaldrich.cn/CN/zh/product/sigma/hpa064616
3. TEAD4 (NBP1-32765) https://www.novusbio.com/products/tead4-antibody_nbp1-32765
4. Anti-TEAD4 (ab58310) https://www.abcam.com/products/primary-antibodies/tead4-antibody-5h3-ab58310.html
5. YAP (sc-101199) https://www.scbt.com/p/yap-antibody-63-7/
6. WWTR1 (CL488-66500) https://www.ptglab.co.jp/Products/TAZ-Antibody-CL488-66500.htm
7. beta Actin (sc-47778) https://www.scbt.com/zh/p/beta-actin-antibody-c4/
8. Oct3/4 (sc-5279) https://www.citeab.com/antibodies/816927-sc-5279-anti-oct-3-4-antibody-c-10
9. SOX2 (AF2018) https://www.rndsystems.com/cn/products/human-mouse-rat-sox2-antibody_af2018
10. Anti-Nanog (ab21624) https://www.abcam.com/nanog-antibody-ab21624.html
11. Anti-alpha Tubulin (ab7291) https://www.abcam.com/products/primary-antibodies/alpha-tubulin-antibody-dm1a-loading-control-ab7291.html
12. Anti-Histone H3 (ab1791) https://www.abcam.com/histone-h3-antibody-nuclear-marker-and-chip-grade-ab1791.html
13. Anti-Histone H4 (ab10158) https://www.abcam.cn/histone-h4-antibody-chip-grade-ab10158.html
14. Anti-acetyl-Histone H3 (06-599) https://www.sigmaaldrich.cn/CN/zh/product/mm/06599
15. Histone H4ac (39926) https://www.thermofisher.com/antibody/product/Histone-H4ac-pan-acetyl-Antibody-Polyclonal/39926
16. Anti-Histone H3 (acetyl K27) (ab4729) https://www.abcam.com/histone-h3-acetyl-k27-antibody-chip-grade-ab4729.html
17. AP-2γ (sc-12762) https://www.scbt.com/p/ap-2gamma-antibody-6e4-4/
18. GATA3 (sc-268) https://www.scbt.com/zh/p/gata-3-antibody-hg3-31
19. KLF17 (HPA024629) https://www.sigmaaldrich.cn/CN/zh/product/SIGMA/HPA024629
20. Anti-HLA G (ab283260) https://www.abcam.cn/products/primary-antibodies/hla-g-antibody-epr23298-97-ab283260.html
21. Anti-SDC1 (ab39969) https://www.abcam.cn/products/primary-antibodies/scd1-antibody-ab39969.html
22. IgG (A7058) https://beyotime.com/product/A7058-2mg.htm
23. IgG (A7050) https://www.beyotime.com/product/A7050-2mg.htm
24. Alexa Fluor 488-AffifiniPure Donkey Anti-Mouse IgG (H+L) (715-545-150) https://www.jacksonimmuno.com/catalog/products/715-545-150
25. Alexa Fluor 488-AffifiniPure Donkey Anti-Rabbit IgG (H+L) (711-545-152) https://www.jacksonimmuno.com/catalog/products/711-545-152
26. Cy3-AffifiniPure Donkey Anti-Rabbit IgG (H+L) (711-165-152) https://www.jacksonimmuno.com/catalog/products/711-165-152
27. Goat anti-Rabbit IgG (H+L) Secondary Antibody, HRP (31460) https://www.thermofisher.cn/cn/zh/antibody/product/Goat-anti-Rabbit-IgG-H-L-Secondary-Antibody-Polyclonal/31460
28. Goat anti-Mouse IgG (H+L) Secondary Antibody, HRP (31430) https://www.thermofisher.cn/cn/zh/antibody/product/Goat-anti-Mouse-IgG-H-L-Secondary-Antibody-Polyclonal/31430

Eukaryotic cell lines

Policy information about cell lines and Sex and Gender in Research

Cell line source(s)

HEK293T were purchased from ATCC. Human H9 ESCs (WA09) were purchased from WiCell Research Institute, human UH10 iPSCs were provided by Dr. G. Pan (Guangzhou Institutes of Biomedicine and Health, Chinese Academy of Sciences, China), and blastocyst-derived TSCs were provided by Dr. Hiroaki Okae and Dr. Takahiro Arima (Department of Informative Genetics, Tohoku University Graduate School of Medicine).

Authentication

HEK293T cells were validated by ATCC. We authenticated H9 ESCs by STR analysis.

Mycoplasma contamination

All cell lines were negative for mycoplasm.

Commonly misidentified lines
(See ICLAC register)

To our knowledge, no cell lines used in this study are listed in the database of commonly misidentified cell lines maintained by ICLAC.

Plants

Seed stocks

Not applicable.

Novel plant genotypes

Not applicable.

Authentication

Not applicable.

ChIP-seq

Data deposition

☒ Confirm that both raw and final processed data have been deposited in a public database such as GEO.

☒ Confirm that you have deposited or provided access to graph files (e.g. BED files) for the called peaks.

Data access links
May remain private before publication.

https://www.ncbi.nlm.nih.gov/geo/query/acc.cgi?acc=GSE193621

Files in database submission

TELC-D5.H3K27ac_rep1
TELC-D5.H3K27ac_rep2
TELC-D5.H3K27ac_rep3
TSC.H3K27ac_rep1
TSC.H3K27ac_rep2
TSC.H3K27ac_rep3
4CL.H3K27ac_rep1
4CL.H3K27ac_rep2
4CL.H3K27ac_rep3
TELC-D5.TEAD4_rep1
TELC-D5.TEAD4_rep2
TSC.TEAD4_rep1
TSC.TEAD4_rep2
4CL.TEAD4_rep1
4CL.TEAD4_rep2
TELC-D5.VGLL1_rep1
TELC-D5.VGLL1_rep2
TSC.VGLL1_rep1
TSC.VGLL1_rep2
WT_TELC-D5.H3K27ac_rep1
WT_TELC-D5.H3K27ac_rep2
WT_TELC-D5.TEAD4_rep1
WT_TELC-D5.TEAD4_rep2
C68_TELC-D5.H3K27ac_rep1
C68_TELC-D5.H3K27ac_rep2
C68_TELC-D5.TEAD4_rep1
C68_TELC-D5.TEAD4_rep2
TELC-D5.YAP_rep1
TELC-D5.YAP_rep2
TELC-D5.GATA3_rep1
TELC-D5.GATA3_rep2
TELC-D5.TFAP2C_rep1
TELC-D5.TFAP2C_rep2

Genome browser session
(e.g. UCSC)

Integrative Genomics Viewer (IGV)

Methodology

Replicates

Samples have two replicates: TELC-D5.VGLL1, TELC-D5.TEAD4, TSC.VGLL1, TSC.TEAD4,
WT_TELC-D5.H3K27ac, WT_TELC-D5.TEAD4, C68_TELC-D5.H3K27ac, C68_TELC-D5.TEAD4,
TELC-D5.YAP, TELC-D5.GATA3, TELC-D5.TFAP2C

Samples have three replicates: TELC-D5.H3K27ac, 4CL.H3K27ac, TSC.H3K27ac

Sequencing depth	50 million pair-end raw reads for each sample with the length of 150bp.
Antibodies	VGLL1 antibody, Invitrogen, Cat# PA5-59968<br>TEAD4 antibody, Novus, Cat# NBP1-32765<br>Anti-Histone H3 (acetyl K27) antibody, abcam, Cat# ab4729<br>YAP antibody, Santa Cruz Biotechnology, Cat# sc-101199<br>GATA3 antibody, Santa Cruz Biotechnology, Cat# sc-268<br>AP-2γ antibody, Santa Cruz Biotechnology, Cat# sc-12762
Peak calling parameters	Peaks were called using MACS2 (v2.1.0) with the settings "-B --nomodel --keep-dup 1 -g hs --call-summits -q 1e-05"
Data quality	Samples with Q30 >85% were used for analyses. Raw reads were then trimmed of adapters and low-quality reads were removed by fastp (v0.21.0) with the default option. Overall alignment rate of all samples were over 90%. Biological replicates with Pearson coefficient > 0.8 were retained for further analyses.
Software	fastp (v0.21.0), Bowtie2 (v2.2.5), Samtools (v1.10), Picard (v1.9.0), MACS2 (v2.1.0), clusterProfiler (v3.6.0), ChIPseeker (v1.26.2), ChIPpeakAnno (v3.24.2), deeptools (v3.4.3), ggplot2 (v3.3.3).
