# Supplementary material for: Author Correction: VGLL1 cooperates with TEAD4 to control human trophectoderm lineage specification
Source: Nat Commun. 2025 Mar 18;16:2643. doi: 10.1038/s41467-025-57929-w (PMC11920433; doi:10.1038/s41467-025-57929-w)

| | Initially published | Author correction |
|---|---|---|

## 1)

**Initially published**

**SFig.3h**

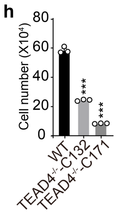


SFig.3n

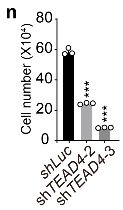


Source data of SFig.3h was correct.

**Author correction**

**SFig.3h**

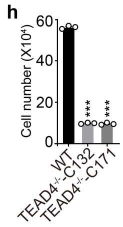


**Initially published**

**SFig.3q**

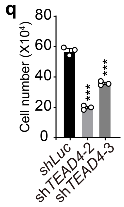


SFig.2h

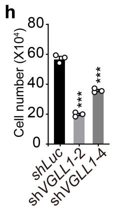


Source data of SFig.3q was correct.

**Author correction**

**SFig.3q**

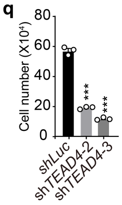


## 2)

**Initially published**

**SFig.4h**

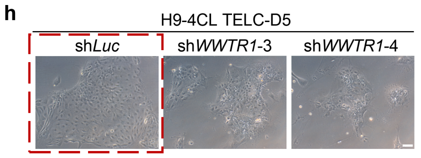


SFig.3p

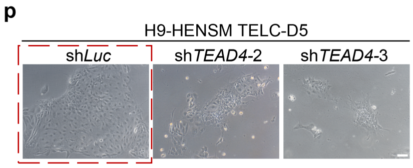


**Author correction**

**SFig.4h**

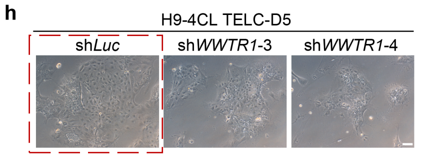


**Initially published**

**SFig.7v**

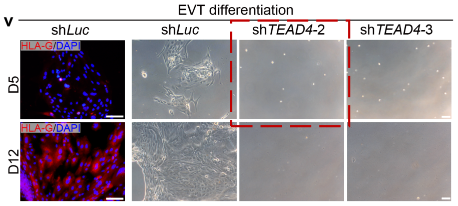


SFig.7w

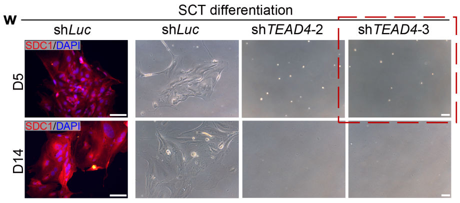


**Author correction**

**SFig.7v**

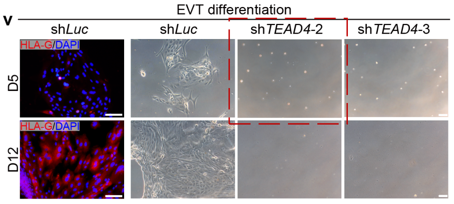


## 3)

**Initially published**

**SFig.8m**

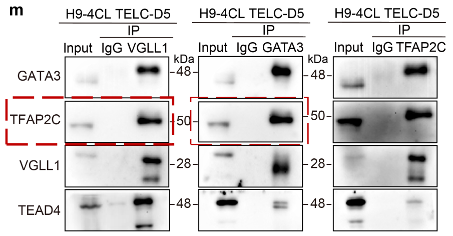


**Author correction**

**SFig.8m**

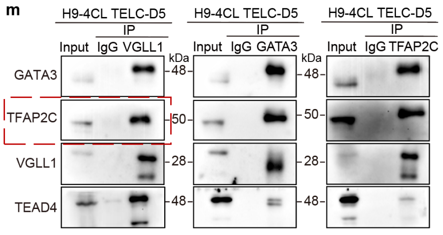


**Initially published**

**SFig.9**
(Uncropped scans)

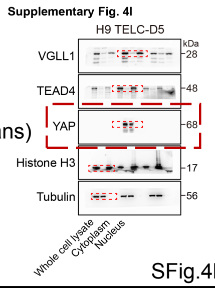


SFig.4l

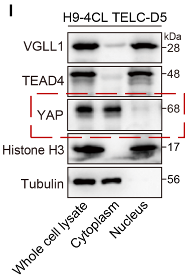


SFig.4l was correct.

**Author correction**

**SFig.9**
(Uncropped scans)

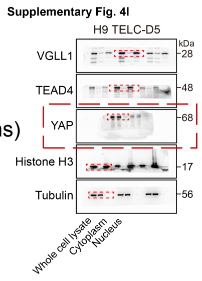


**Initially published**

**SFig.9**
(Uncropped scans)

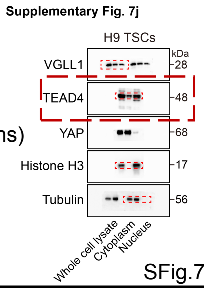


SFig.7j

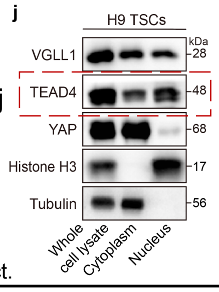


SFig.7j was correct.

**Author correction**

**SFig.9**
(Uncropped scans)

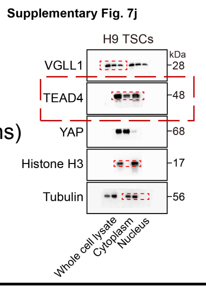

Supplement: Supplementary file 1 — Supplementary information [file 41467_2025_57929_MOESM1_ESM.pdf]
